# Flake Graphene-Based Nanomaterial Approach for Triggering a Ferroptosis as an Attractive Theranostic Outlook for Tackling Non-Small Lung Cancer: A Mini Review

**DOI:** 10.3390/ma15103456

**Published:** 2022-05-11

**Authors:** Joanna Pancewicz, Wiesława Ewa Niklińska, Adrian Chlanda

**Affiliations:** 1 Department of Histology and Embryology, Medical University in Bialystok, Waszyngtona 13, 15-269 Białystok, Poland; joanna.pancewicz@umb.edu.pl (J.P.); wieslawa.niklinska@umb.edu.pl (W.E.N.); 2 Graphene and Composites Research Group, Łukasiewicz Research Network—Institute of Microelectronics and Photonics, al. Lotników 32/46, 02-668 Warszawa, Poland

**Keywords:** flake graphene, graphene oxide, ferroptosis, non-small lung cancer, nanomaterials

## Abstract

Lung cancer is a highly aggressive neoplasm that is now a leading cause of cancer death worldwide. One of the major approaches for killing cancer cells is related with activation of apoptotic cell death with anti-cancer drugs. However, the efficiency of apoptosis induction in tumors is limited. Consequently, the development of other forms of non-apoptotic cell death is up to date challenge for scientists worldwide. This situation motivated us to define the aim of this mini-review: gathering knowledge regarding ferroptosis—newly defined programmed cell death process characterized by the excessive accumulation of iron—and combining it with yet another interesting nanomaterial-based graphene approach. In this manuscript, we presented brief information about non-small lung cancer and ferroptosis, followed by a section depicting the key-features of graphene-based nanomaterials influencing their biologically relevant properties.

## 1. Non-Small Lung Cancer

Cancer is described as a group of disorders entailing abnormal cell growth with the ability to spread to other organs and tissues in the body. Human lung cancers are divided into small cell lung cancer (SCLC) and non-small cell lung cancer (NSCLC), which originates from epithelial cells. NSCLC is very heterogeneous group of cancers and is composed of different histological subtypes including mainly squamous-cell carcinoma, large-cell carcinoma, and adenocarcinoma [1]. Moreover, lung cancer is highly aggressive and challenging neoplasm, with an established 5-years survival rate under 20% [2,3]. At the same time, it should be mentioned that nearly 85% of all diagnosed lung cancers are covered with non-small cell lung cancer cases [4].

Although, currently used anti-cancer therapies including chemo- and radiotherapy have prolonged overall survival among patients, other challenges, such as metastasis and chemoresistance, emerged. These are, up until no, one of the biggest clinical problems that are receiving great attention regarding clinical oncology [5,6]. Currently, the treatment of choice in case of NSCLC is radical surgery; however, only 20–25% of diagnosed patients were allowed to be treated with surgical approaches [7]. Adjuvant cisplatin-based chemotherapy is the gold standard for fully resected NSCLC tumors [8]. At the same time, new generations of drugs that target a specific genes mutations or protein have been approved as personalized treatments in NSCLC patients [9].

A lack of early symptoms and effective early diagnostic methods results in a diagnosis of cancer often in an inoperable, advanced stage of the disease. Therefore, there is an urgent need for novel, effective diagnostic methods and therapies for lung cancer. Activating apoptotic cell death with anti-cancer drugs is one of the major approaches for killing cancer cells. Nevertheless, the efficiency of apoptosis induction in tumors is limited due to the acquired or intrinsic resistance of cancer cells relative to apoptosis [10,11]. Consequently, developing other forms of non-apoptotic cell death opens new therapeutic opportunities for eliminating cancer cells and limiting the survival of drug-resistant clones.

Regarding lung-cancer therapy, graphene was already considered as an attractive material for screening (biosensing) or direct anti-cancer treatment. An interesting approach was presented in a paper written by Chen et al. [12], in which graphene with silver particles was used to fabricate simple, highly sensitive and fast biosensor. This biosensor was calibrated to detect CYFRA21-1 gene of NSCLC patients and was successfully tested during clinical trials, proving that graphene can be considered as a material for the selective and sensitive detection of DNA. Hence, it is possible to introduce graphene-based sensors as an efficient tool for the early diagnosis of NSLC.

In addition, graphene flakes conjugated with cisplatin (a chemotherapy agent used to treat a number of cancers) were confronted against the non-small lung carcinoma A549 cell line [13]. Using this nanomaterial-based approach, the authors of this manuscript tried to implement graphene flakes as an efficient drug-delivery platform, from which a highly concentrated dose of a medicine could be released. Based on the obtained results, it was concluded that drug-delivery systems including graphene can be considered as a remedy for various types of cancer.

For the same cell line, A549 carcinoma lung cells were a part of Zuchowska et al.’s study, in which the authors described submicrometric graphene oxide (GO) flakes—one of the flake graphene derivatives, as a promising material designed for lung cancer therapy [14]. It was stated that anticancer feature of GO flakes was triggered by a reactive oxygen species at high concentrations (>300 µg/mL) of such nanomaterial. Moreover, the affinity of graphene flakes to A549 cell nucleus was observed, which resulted in decreased cellular proliferation.

Having all this in mind, it can be concluded that GO flakes were successfully implemented as anticancer agents for effective NSLC therapy.

## 2. Ferroptosis

Reactive oxygen species (ROS) [15,16] along with excessive accumulation of iron is one of the ferroptosis pivotal mechanisms. It is worth underlining that ferroptosis is a newly defined programmed cell death process. It is recognized as an efficient mechanism to eliminate malignant cells. It plays an essential role in the inhibition of oncogenic processes by removing cells that are not able to keep nutrients in the environment or cells damaged by infection or stress [9]. Moreover, ferroptosis occurs together with defeated of repair system that are responsible for the removal of lipid peroxidation products in physiological homeostatic conditions. Contrary to healthy cells, cancer cells in majority lack repair systems, which means that they are more vulnerable to oxidative damage and, thus, ferroptosis.

Nevertheless, the natural function of ferroptosis in physiological contexts is still not clear. There are some ideas that ferroptosis may serve as a normal tumor suppressive function, including the observation that tumor suppressors p53, fumarase, and BAP1 can drive ferroptosis under specific conditions, and that some negative regulators of ferroptosis, such as SLC7A11, GPX4, and NRF2 are overexpressed or activated in a diversity of tumors [17,18].

**Figure 1:**
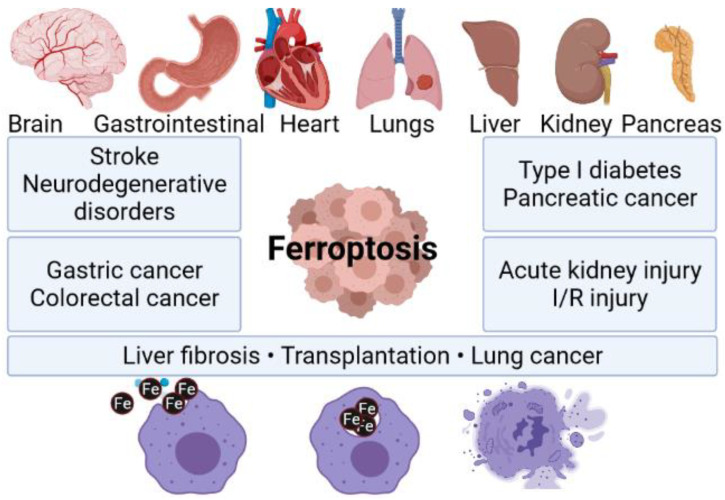
Ferroptosis as a factor triggering failure of multiple organs. Composed with BioRender [21].

It is worth mentioning that ferroptosis can be responsible for a failure of different crucial organs, including, i.e., lungs, heart, or brain (Figure 1). For instance, one can find scientific articles describing ferroptosis-dependent neural diseases, including the following: cerebral stroke, Parkinson’s, or Alzheimer’s disease. Having this in mind, ferrotosis can be considered as an unwanted and dangerous factor affecting human health. However, if properly designed and controlled (using nanomaterial-based approach), it can be also be a useful tool for targeted anticancer remedies. It is well known that tumor cell death is the most desirable effect of any cancer therapy. However, similarly to what was mentioned earlier, available anticancer drugs directed to kill cancer cells through apoptosis have limited effects since acquired resistance to apoptosis occurs [19]. Therefore, the main goal of ferroptosis-based therapy in cancer is to prevent treatment resistance. Unlike other forms of cell death, ferroptosis is iron- and ROS-dependent. To date, several mechanisms on which ferroptosis can act in tumor cells have been indicated. Researchers described in great detail how the resistance to targeted cancer therapy could be reversed by inducing ferroptosis through iron and lipid metabolism pathways, as well as other signaling pathways. A perfect example of new ferroptosis-based therapeutic strategy in NSCLC is the overcoming of cisplatin resistance within NF-E2 related factor 2 (Nrf2)/light chain of System xc−(xCT) pathway given by Yu Li et al. [20]. Moreover, it has been recently presented that the inhibition of ferroptosis is engaged in cancer immunotherapy by anti-programmed cell death 1 (PD-1)/programmed death-ligand 1 (PD-L1) treatment resistance [9].

Off these efforts, nanotechnology-based methods are especially remarkable, given their theragnostic approaches. Understanding the specific mechanism of ferroptosis and its relationship with lung cancer could provide significant references regarding cancer therapy [22,23,24]. It is well accepted that the occurrence of ferroptosis is iron-dependent and it can be assumed that, i.e., thoughtfully designed graphene oxide [25,26] (very promising 2D carbon-based materials for possible biomedical application) modifications with iron can be used as a new system to induce ferroptosis. Such tailor-made graphene oxide-Fe (graphene oxide-iron) composite could be a good option to induce and further study not only ferroptosis but other molecular changes in cancer cells after extracellular Fe intake.

Graphene-based nanomaterials such as as a ferroptosis-inducing agents were already considered by other authors, who paid special attention to graphene quantum dots (GQDs). GQDs are quantum dots smaller than 20 nm and composed of carbon atoms. Two types of GQDs were studied by Wu et al. [27], namely amino-functionalized and nitrogen-doped graphene dots. The authors concluded that nitrogen-doped GQDs induced ferroptosis by mitochondrial oxidative stress. This phenomenon was associated with the disruption of a morphology of mitochondria, which was additionally translated to redox imbalance and iron overload. Further investigation performed by the same research team bore fruit in the form of even more detailed descriptions of GQDs-triggered ferroptosis mechanism [28]. The authors stated that key factors responsible for graphene quantum dots ferroptic action is the disruption of calcium homeostasis in microglia. Interestingly, BV2 (microglial cell line) cells exposed to amino-functionalized GQDs were less prone to oxidative stress, which indicate that the surface modification of graphene-based materials can be of crucial importance for triggering specific cellular responses.

**Figure 2:**
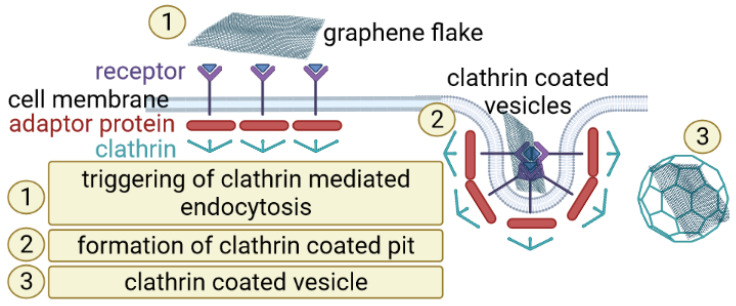
A scheme of clathrin-mediated graphene endocytosis. Composed with BioRender [21].

Understanding the entry mechanism of graphene derivatives relative to cells would be significant for the evaluation of its interaction within cells and further clinical application. Endocytosis, an energy-dependent mechanism, is known to be the entry mechanism of graphene [29]. Experimental studies have suggested that, due to GO 2D structure, it could be taken up by cancer cells via clathrin-mediated endocytosis (Figure 2).

However, another hypothesis is that graphene-based materials are able to penetrate and disrupt the cell membrane mostly thanks to their sharp edges (acting as nano-sized knifes) [30]. Such nano-knife can damage cell membrane and cause the leakage of phospholipids from cell body. A huge number of scientific publications were written in order to address the biomedical application of graphene family materials, and to date, contradictory arguments are present in this discussion [29,31,32,33]. The possible explanation could be that the current state of knowledge and scientific tools are not sufficient to fully understand and describe complex biological interactions occurring on the graphene–living cell interface.

## 3. Nanomaterial Based Ferroptosis Inducers in NSCLC

A literature analysis of ferroptosis inducers (including small molecules and nanomaterials) is presented to define their design, action mechanisms, and anticancer applications; however, there are still many questions about mechanisms governing the killing activity of cancer cells through ferroptosis and its implications at molecular levels. The mechanisms of action of apoptosis or necroptosis are reliant on caspases or can be inhibited by their activity. It should be mentioned that ferroptosis appears to have advanced independent and very little known yet direct molecular cross-talk to other pathways of regulated cell death [34]. Specially designed nanomaterials (NMs) are known to be able to penetrate the human body through respiratory systems, oral ingestion, or a skin. Furthermore, NMs are capable to cross the plasma membrane in order to start cell death processes. Nanoparticles are considered as a novel ferroptosis inducer with possible therapeutic effects in a wide range of cancer types, including NSCLC. It is widely known that the distraction of cell-death homeostasis is related to various diseases, including neurodegenerative diseases, immune disorders, diabetes, and cancer. Available data indicate that iron- or iron-oxide-based NMs can be considered as a good approach to study ferroptosis and its implications in cancer. Mechanisms of iron-based NPs and non-iron NPs for ferroptosis-based cancer therapy are similar. Both types of NPs can be incorporated into cells through endocytosis and release iron in lysosomes. Furthermore, they can be involved in the Fenton reaction to produce reactive oxygen species (ROS) and, as a consequence, induce ferroptosis. Moreover, the drugs carried by nanoparticles can facilitate the production of ROS, which is caused by excess iron. β-lapachone (β-lap), a novel anticancer drug, has shown significant cancer specificity by increasing (ROS) stress in cancer cells. A 10-fold increase in ROS stress was detected in β-lap-exposed cells pretreated with iron oxide nanoparticle over cells treated with β-lap alone in A549 cells, which also correlates with significantly increased cell death [35].

Regardless of the nanomaterials’ size and origin, the overriding conclusion drawn by the authors of the manuscripts provided in Table 1 was that this theranostic approach is very promising. The results of their work clearly showed that nanomaterials can be implemented as an efficient tool for anticancer therapy. Interestingly, different types of cancer models were investigated, including inter alia breast, lung, colorectal, cervical, pancreatic, colon, and leukemia. All of the cited publications underlined that the utilization of nanoparticles enabled the triggering of ferroptosis. Hence, it can be hypothesized that nanomaterials can be considered as a future cancer remedy. However, a lot of work still has to be performed in order to fully understand and describe underlying ferroptosis mechanisms activated by nanomaterials.

We want to underline that the main advantage of graphene-based system over other nanomaterials-based ferroptosis induction systems is the fact that graphene can be treated as a tailor-made material suited for a very specific applications—in the scope of this manuscript, toward NSLC therapy. Having said this, it is possible to design and synthesize graphene flakes (GO, RGO, or GQDs) of a given size (lateral dimensions in some specific range), provide alevel of reduction (translated to modification feasibility), and to modify them in order to add desired functionality—such as ferroptosis induction. By performing this, it will be possible to deliver a highly concentrated dose of iron particles to cancerous tissue. What is more is that, as stated earlier, GO flakes itself can also promote ferroptosis; thus, by a combination of GO flakes with Fe nanoparticles, synergistic effects aimed to fight against NSLC could be achieved.

## 4. Physical and Chemical Properties of Flake Graphene-Based and Other Nanomaterials—The Impact of Shape, Size, and Large Specific Surface on Graphene Biological Effect

The graphene oxide (GO) production protocol is well described in the literature and is referred as Hummer’s method, alongside its multiple modifications [46,47]. In brief, GO synthesis is based on mixing graphite materials with strong oxidizers, such as fuming nitric acid or potassium chlorate. Using this method, it is possible to introduce oxygen atoms into closely stacked graphite layers. The successful implementation of Hummer’s method results in a single atomic layer of GO. Such 2D carbon sheet is characterized with a sp2 hybrid structure embroidered with hydrophilic surface groups. The presence of these hydrophilic groups is needed to constitute stable water solutions of GO flakes. After thermal [48] or chemical treatment [49], GO flakes undergo reduction, and the amount of oxygen-based functional groups decreases, and a new form of material is created, namely RGO flakes. It is important to know that GO reduction is followed by a change in the material’s properties. RGO is described as a conductive yet hydrophobic material, which governs different possible mechanisms of cell–material interactions and, thus, different applications.

**Figure 3:**
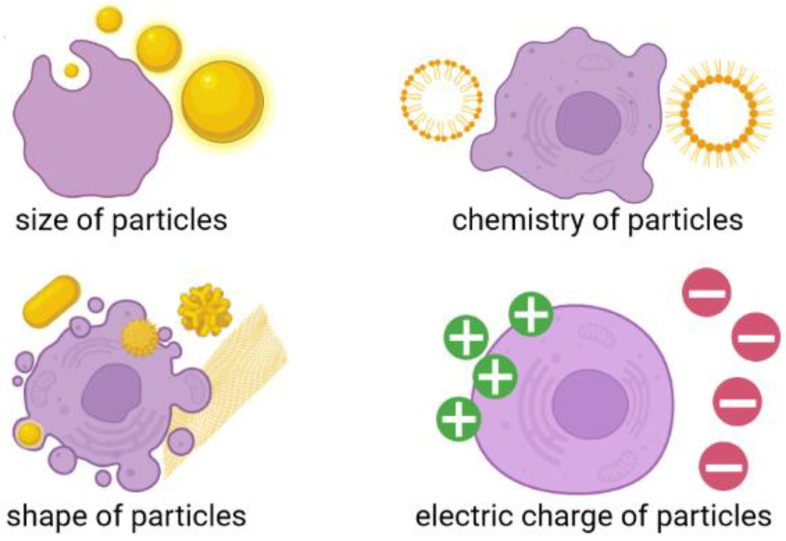
Different factors affecting cell-nanomaterial interaction. Composed with BioRender [21].

Although flake graphene and its derivatives are considered as very promising materials that are described by conductive (depending on reduction level) and super-elastic properties, with a projected potential for manifold applications in the biomedical field, the safety of its production, utilization, and potential toxicity is still not examined and understood. This is mostly driven by the nanometric size of these peculiar carbon materials. Many efforts have been made to fulfill this gap; however, detailed toxicity evaluation of both GO and RGO flakes—especially in the long-time perspective—is still missing. A vast majority of the studies were focused on the assessment of GO toxicity during short periods of time; surprisingly, long-term studies over toxicity influences relative to GO flakes were not taken into account [50,51,52,53]. Another important issue related with this issue is that although the surface modification of GO with materials of different origin (such as nanoparticles, drugs, and polymers) [54] can improve its biocompatibility, the stability of its later deposition on different surfaces is still unknown [55]. This parameter should be considered regarding the design and production of high quality flake graphene-based material tailored for some strictly defined, cutting-edge anti-cancer therapies. Without the estimation of long-term stability of GO flakes [26] and GO-based covering layers, the durability and usability of any novel GO-based biomedical tool will be secreted, which in turn will greatly impede its possible biomedical application. Having this in mind, it can be assumed that, the toxicity of graphene-based materials can be different from currently reported short-term exposure results. In addition, it can also differ when graphene-based coating applied on biomedical device break. Additionally, many studies describing GO flakes toxicity assessment have been already conducted [12], but the number of samples and characterization methods did not provide a clear statement on graphene toxicity. This was mostly driven by the fact that the vast majority of recent studies focused exclusively on GO size or chemical composition and their impact on biological response alone. It should be noted that an approach limited to only one or two features will significantly impede the process of explaining mechanisms affecting living cells. We want to remind the reader that a certain biological function or biological response can be driven by multiple factors, such as the size, shape, chemistry, and electrical charge of nanomaterials (Figure 3).

At the current time, two polarizing opinions on GO toxicity have emerged: Some researchers hypothesized that GO is biocompatible [14] whereas other studies reported negative biological responses and cytotoxic effects caused by GO and RGO flakes [56,57]. In the seminal work of Jagiełło et al. [58], the authors made a step forward in understanding the relations between two important factors (size and reduction level) of graphene oxide flakes and biological response, determined upon human umbilical cord Wharton’s jelly-derived mesenchymal stem cells (hUC-MSCs). It should be underlined that both GO and RGO flakes were suitable materials for the proliferation of hUC-MSCs. Cells cultured on a thin layer of GO and RGO (of low reduction level) were characterized with a viability and proliferation rate similar to standard culture conditions (without the presence of graphene flakes). Hence, no toxic reaction, driven by graphene flakes, was observed. Moreover, reduced GO flakes triggered cell apoptosis. Another interesting outcome of this study was that, regardless of the reduction level and size of the graphene flakes, hUC-MSCs showed no alterations of their phenotype. Hence, the authors concluded that GO flakes and RGO flakes with low reduction levels were not toxic and could be taken into consideration regarding possible applications toward regenerative medicine.

The general finding from the basis of all aforementioned literature results is that graphene oxide-Fe based modifications can be developed as a novel method to induce cancer cell death through ferroptosis, possibly with lysosomes, and improve intracellular Fenton reactions to produce ROS and induce lipid peroxidation. This statement is greatly motivated by recently published work where other types of graphene material—graphene quantum dots—were used to induce ferroptosis in microglia [15].

Once more, we want to point out that several factors influencing the toxicity of graphene-based materials (and other nano-sized materials) should be taken into account and be systematically studied in order to understand how desired biological functions can be triggered. These properties include, but are not limited to, the following: concentration, lateral dimension, surface structure, and functionalization of flake graphene. Thus, it is of crucial importance to perform not only systematic but also statistically significant screening of different kinds of any nano-sized material, including both GO and RGO flakes [59]. For instance, an interesting study related with iron nanoparticles uptake by living cells was described by Dulińska-Molak et al. [60]. In this study, three types of carbon-encapsulated iron nanoparticles were synthesized, and their impact of the mechanical properties of human mesenchymal stem cells (hMSCs) was assessed by means of atomic force microscopy. The authors noticed that the concentration-related dependence of the stiffness of cells exposed to iron-based nanoparticles. The higher the nanoparticles concentration, the lower the registered stiffness of the cells. The overriding conclusion drawn by this study was that iron particles deprived of carbon coating were the most lethal and even low concentrations of such particles resulted in an alteration of hMSCs actin cytoskeleton.

**Figure 4:**
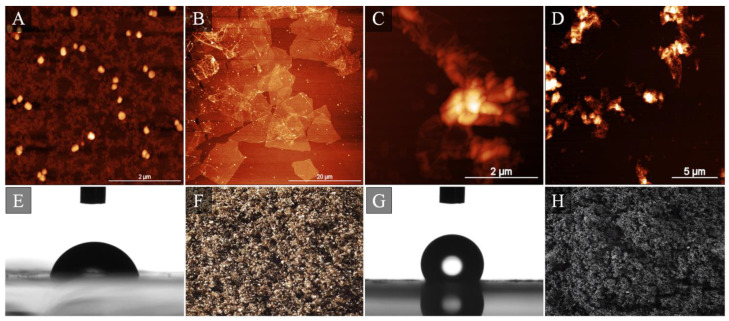
Representative atomic force microscopy images of GO nano- (**A**) and micrometric (**B**) flakes along with RGO flakes (**C**,**D**). Images (**E**,**G**) present water contact angle results acquired for hydrophilic GO and hydrophobic RGO followed by optical images of GO (**F**) and RGO (**H**) powder. Image composed with Authors’ own and previously not published data.

Another interesting example of multi-technique-based approaches toward in-depth descriptions of interactions between cells and nanomaterial was a study written by Oberbek et al. [59] in which in vitro studies of manifold forms of hydroxyapatite (HAp) nanoparticles were addressed. The authors characterized 10 different types of HAp nanoparticles describing inter alia morphology, average particle size, particle shape expressed by aspect ratio, specific surface area, state of agglomeration, density, crystallinity, phase purity, stoichiometry, zeta potential, and pH. They then confronted their results against four cell lines by imitating different systems present in human body: Chinese hamster ovary cell line (reproductive system), mouse monocyte macrophage cell line (immune system), human bronchial epithelial cell line, and human lung adenocarcinoma epithelial cell (respiratory system). They concluded that “the biological impact depends on dose and physicochemical properties of the HAp particles and the cell nature”. Hence, it is of prime importance to conduct systematic and multi-technique studies for any nanomaterial, including graphene flakes (Figure 4).

However, there are other alternative factors that need to be discussed in the context of GO treatment of cancer, which depend, for example, on the vascularization of the tumor and its microenvironment in general. Contrary to the normal vascular system, which is resistant to some bigger particles, tumors have porous vascular systems, which are more vulnerable to the uptake of different extracellular molecules into the cell. Moreover, cancer diminishes the function of lymphatic systems, making it easier for nanoparticles to remain in the tumor’s environment [61]. In addition, when considering graphene compounds in cancer therapy, the type of cells and origin should be kept in mind. Zuchowska A et al. showed that liver cells (either cancer and non-cancerous) were more sensitive to the tested graphene compounds than compared to breast cells. The authors pointed a couple of reasons for the above differences, including morphological and functional variations [62]. Other studies indicated that graphene-based nanomaterials could influence cancer stem cells via various signaling pathways to differentiate them into non-cancerous stem cells [63].

## 5. Possible Adverse Effects Triggered by Graphene Flakes and Other Nanomaterials

Although GO and RGO flakes and other nanomaterials are very promising materials regarding their biomedical applications, there are still some serious challenges on the horizon to be tackled and size-related issues to be addressed prior to a fully controllable bio-implementation of nanomaterials.

Thus, it is of prime importance to understand any material-cells and material-tissues interactions that may appear upon material uptake. First, it should be noted that GO and RGO flakes can be transferred to human body using different exposition routes, including inhalation, oral, or skin administration. Due to its small size, flake graphene can easily penetrate blood–brain [64] or blood–air barriers and then accumulate in the internal organs, such as spleen or liver. Interesting, a study was provided by Su and coworkers [65] who designed an experiment in order to assess the deposition of GO flakes in human upper airways. They tested flakes characterized average lateral sizes of 51, 101, and 215 nm; thus, they were very small flakes with high potentials to penetrate cells and tissues. Regardless of the size of tested flakes, the deposition of nanometric carbon-based material in airway sections was smaller than 4%. The authors concluded that most of the studied flakes could be transferred to the lower tracheobronchial airways. Moreover, it was speculated that such situations could trigger some health issues. Several publications have already outlined toxicological mechanisms of graphene-based nanomaterials, including inter alia apoptosis, necrosis, and inflammation [66,67,68,69].

A seminal work written by Zhang et al. [70] was devoted to the assessment of biocompatibility of GO flakes using mice animal model exposed to intravenous administration of nanomaterial. The GO flakes implemented in this study were smaller than 800 nm, with a thickness of 1 nm. A radiotracer technique was used to examine uptake and the retention of GO flakes. The authors reported that GO flakes were mostly deposited in lungs and stayed there for a long time. Interestingly, when compared to different carbon nanomaterials, GO was characterized with long circulation time in blood (of half-time in the range 5.3 ± 1.2 h). Of special interest was the fact that any pathological changes were characterized in a GO dose-dependent manner. Small dosage (1 mg kg^−1^ of mice body weight) did not trigger any pathological changes during 14 days of exposition. It was concluded that such results guided GO applications toward drug delivery, especially to the lung. On the other hand, the authors have noticed pathological changes when higher GO flake amounts (10 mg kg^−1^ of mice body weight) were used. These changes were related with cellular inflammation and granuloma formation in the after 14 days of exposition to flakes. Such conclusions were actually expected, as an overdose of any material would cause similar effects. Graphene-based nanomaterials are no exception in this manner. However, once again, we want to underline that, in the case of graphene flakes, it is important to remember other parameters influencing their final application. GO and RGO flakes can have both positive (tissue regeneration and drug delivery systems) and negative (cellular apoptosis and inflammation) effects on the human body. It does not mean, however, that each graphene-based material is dangerous to human health. The challenge here is to select an appropriate set of material’s parameters and to have the control over purity and reproducibility of the designed graphene-based material. By using a proper combination of size, concentration, level of oxidation, and chemical modifications, it is possible to design and fabricate graphene-based biomedical tool of certain functions, which will trigger desired biological answers. Similar conclusions were proposed by Fadeel and coworkers [71], who additionally questioned whether or not is possible to predict the adverse (toxic) influence of graphene-based materials on living organisms by focusing only on their properties, thus omitting in vitro and in vivo studies. One thing can be treated as an axiom—further studies and scientific scrutiny are needed to fully address complex graphene–living organism interactions.

## 6. Conclusions and Future Outcomes

One of the still up to date and important scientific challenges to tackle is to understand the subtle interactions occurring on the interface of biological materials (living cells) and laboratory-fabricated materials, such as graphene flakes. If this is successful, this might be the key opening the door for the synthesis and fabrication of 2D graphene-based nanomaterial tools to fight against non-small lung cancer and hopefully other types of cancer as well. Graphene oxide, along with its functionalized alter ego already showed promising results towards biomedical applications. At the same time, it should be emphasized that, due to te two competitive positions regarding the biological evaluation of GO and RGO materials, further scientific scrutiny is needed to fully explore of this specific terra incognita. We believe that the current scientific apparatus may not be sufficient to fully expose and understand a complex nano- and micrometric mechanisms governing cell–material interactions. At the same time, it should be mentioned that current polarization regarding GO and RGO toxicity may be driven by the fact that different studies addressing this subject introduced different types of graphene in terms of size, shape, chemical purity, surface modification, and synthesis protocol. This in turns yields different cellular responses. A combination of a better standardization of graphene flakes per se along with the implementation of the newest scientific tools for materials characterization can be a foundation of a new, biomedical graphene era.

## Figures and Tables

**Table 1:** Nanoparticle-based approach aimed to trigger ferroptosis.

Nanoparticle Type	Nanoparticle Size	Cancer Model	References
AIEgen/vermiculite nanohybrid	300 nm diameter, 1.1 nm thickness	MC38 tumor model	[36]
polyvinyl pyrrolidone dispersed nanoscale metal-organic framework of Fe-TCPP (TCPP = tetrakis (4-carboxyphenyl) porphyrin) loaded with hypoxia-activable prodrug tirapazamine and coated by the cancer cell membrane	201 nm diameter of the whole system	Breast cancer cells (MDA-MB-231), human liver cancer cells (Huh7, HepG2), human colon cancer cells (HCT116), human pancreatic cancer cells (PATU8988), cervical cancer cells (HeLa)	[37]
Glycyrrhetinic acid loaded PLGA nanoparticles	133 nm in diameter	Leukemia cells: Kasumi-1, U937, MV4–11, NB4, and colorectal cancer cells: HT29, Caco-2, SW480	[38]
Gallic acid-ferrous (GA-Fe(II))	average particle size of 3.1 ± 1.2 nm and hydrodynamic diameter of 4.7 ± 1.1 nm	breast cancer cell (MCF-7/ADR)	[39]
Zinc oxide nanospheres	120 nm in diameter	CT26 and HCT116 colorectal cancer cells	[40]
piperlongumine (PL) loaded metal–organic framework (MOF) coated with transferrin decorated pH sensitive lipid layer	hydrodynamic radius of 185 ± 5.7 nm	4T1 brest cancer cells	[41]
silver coated zero-valent-iron nanoparticles (ZVI@Ag) and carboxymethylcellulose coated zero-valent-iron nanoparticles (ZVI@CMC)	mean physical diameters of ZVI@Ag NPs and ZVI@CMC NPs were 81.08 ± 14.29 nm and 70.17 ± 14.4 nm	Human lung cancer cell lines H1299, H460, A549, mouse Lewis lung carcinoma (LLC)	[42]
Fe(II) and Tannic Acid-Cloaked MOF	125–225 nm	MDA-MB-231 epithelial, human breast cancer cell line	[43]
Manganese doped silica nanoparticle (MnMSN) and folate modified long-circulating MnMSN (FaPEG-MnMSN)	MnMSN—diameter of 101.40 ± 0.36 nm, FaPEG-MnMSN diameter of 122.67 ± 2.98 nm	human hepatic carcinoma cells (HepG2), human non-small lung cancer cells (A549) and mouse breast cancer cells (4T1)	[44]
Superparamagnetic iron oxide nanoparticles (SPION)	99–115 nm of hydrodynamic dimeter	Mouse mammary breast tumor cell line (4T1), human breast cancer cell line (MDA-MB-231) and human breast cancer cell line (MCF-7)	[45]

## References

[1] Pikor L.A., Ramnarine V.R., Lam S., Lam W.L. (2013). Genetic Alterations Defining NSCLC Subtypes and Their Therapeutic Implications. Lung Cancer.

[2] Barta J.A., Powell C.A., Wisnivesky J.P. (2019). Global Epidemiology of Lung Cancer. Ann. Glob. Health.

[3] Ferlay J., Soerjomataram I., Dikshit R., Eser S., Mathers C., Rebelo M., Parkin D.M., Forman D., Bray F. (2015). Cancer Incidence and Mortality Worldwide: Sources, Methods and Major Patterns in GLOBOCAN 2012. Int. J. Cancer.

[4] Shin A., Oh C.M., Kim B.W., Woo H., Won Y.J., Lee J.S. (2017). Lung Cancer Epidemiology in Korea. Cancer Res. Treat..

[5] Iglesias V.S., Giuranno L., Dubois L.J., Theys J., Vooijs M. (2018). Drug Resistance in Non-Small Cell Lung Cancer: A Potential for NOTCH Targeting?. Front. Oncol..

[6] Lee J.S., Hong J.H., Sun D.S., Won H.S., Kim Y.H., Ahn M.S., Kang S.Y., Lee H.W., Ko Y.H. (2019). The Impact of Systemic Treatment on Brain Metastasis in Patients with Non-Small-Cell Lung Cancer: A Retrospective Nationwide Population-Based Cohort Study. Sci. Rep..

[7] Yoon S.M., Shaikh T., Hallman M. (2017). Therapeutic Management Options for Stage III Non-Small Cell Lung Cancer. World J. Clin. Oncol..

[8] Geiger S., Schlemmer M., Heinemann V., Stemmler H.J. (2010). Adjuvant Cisplatin-Based Chemotherapy for Resected NSCLC: One Size Fits All?. Anti-Cancer Drugs.

[9] Jiang W., Cai G., Hu P.C., Wang Y. (2018). Personalized Medicine in Non-Small Cell Lung Cancer: A Review from a Pharmacogenomics Perspective. Acta Pharm. Sin. B.

[10] Holohan C., Van Schaeybroeck S., Longley D.B., Johnston P.G. (2013). Cancer Drug Resistance: An Evolving Paradigm. Nat. Rev. Cancer.

[11] Su N., Wang P., Li Y. (2016). Role of Wnt/β-Catenin Pathway in Inducing Autophagy and Apoptosis in Multiple Myeloma Cells. Oncol. Lett..

[12] Chen M., Wang Y., Su H., Mao L., Jiang X., Zhang T., Dai X. (2018). Three-Dimensional Electrochemical DNA Biosensor Based on 3D Graphene-Ag Nanoparticles for Sensitive Detection of CYFRA21-1 in Non-Small Cell Lung Cancer. Sensors Actuators B Chem..

[13] Augustine S., Prabhakar B., Shende P. (2021). Adsorption of Cisplatin on Oxidized Graphene Nanoribbons for Improving the Uptake in Non-Small Cell Lung Carcinoma Cell Line A549. Curr. Drug Deliv..

[14] Zuchowska A., Jastrzebska E., Mazurkiewicz-Pawlicka M., Malolepszy A., Stobinski L., Trzaskowski M., Brzozka Z. (2019). Well-Defined Graphene Oxide as a Potential Component in Lung Cancer Therapy. Curr. Cancer Drug Targets.

[15] Xu T., Ding W., Ji X., Ao X., Liu Y., Yu W., Wang J. (2019). Molecular Mechanisms of Ferroptosis and Its Role in Cancer Therapy. J. Cell. Mol. Med..

[16] Nie Q., Hu Y., Yu X., Li X., Fang X. (2022). Induction and Application of Ferroptosis in Cancer Therapy. Cancer Cell Int..

[17] Song X., Long D. (2020). Nrf2 and Ferroptosis: A New Research Direction for Neurodegenerative Diseases. Front. Neurosci..

[18] Feng L., Zhao K., Sun L., Yin X., Zhang J., Liu C., Li B. (2021). SLC7A11 Regulated by NRF2 Modulates Esophageal Squamous Cell Carcinoma Radiosensitivity by Inhibiting Ferroptosis. J. Transl. Med..

[19] Mohammad R.M., Muqbil I., Lowe L., Yedjou C., Hsu H.Y., Lin L.T., Siegelin M.D., Fimognari C., Kumar N.B., Dou Q.P. (2015). Broad Targeting of Resistance to Apoptosis in Cancer. Semin. Cancer Biol..

[20] Li Y., Yan H., Xu X., Liu H., Wu C., Zhao L. (2020). Erastin/Sorafenib Induces Cisplatin-Resistant Non-Small Cell Lung Cancer Cell Ferroptosis through Inhibition of the Nrf2/XCT Pathway. Oncol. Lett..

[21] BioRender. https://biorender.com/.

[22] Tao N., Li K., Liu J. (2020). Molecular Mechanisms of Ferroptosis and Its Role in Pulmonary Disease. Oxid. Med. Cell. Longev..

[23] Hao S., Liang B., Huang Q., Dong S., Wu Z., He W., Shi M. (2018). Metabolic Networks in Ferroptosis (Review). Oncol. Lett..

[24] Walters R., Mousa S.A. (2022). Modulations of Ferroptosis in Lung Cancer Therapy. Expert Opin. Ther. Targets.

[25] Chlanda A., Walejewska E., Kowiorski K., Heljak M., Swieszkowski W., Lipińska L. (2021). Investigation into Morphological and Electromechanical Surface Properties of Reduced-Graphene-Oxide-Loaded Composite Fibers for Bone Tissue Engineering Applications: A Comprehensive Nanoscale Study Using Atomic Force Microscopy Approach. Micron.

[26] Chlanda A., Kowiorski K., Małek M., Kijeńska-Gawrońska E., Bil M., Djas M., Strachowski T., Swieszkowski W., Lipińska L. (2021). Morphology and Chemical Purity of Water Suspension of Graphene Oxide FLAKES Aged for 14 Months in Ambient Conditions. A Preliminary Study. Materials.

[27] Wu T., Liang X., Liu X., Li Y., Wang Y., Kong L., Tang M. (2020). Induction of Ferroptosis in Response to Graphene Quantum Dots through Mitochondrial Oxidative Stress in Microglia. Part. Fibre Toxicol..

[28] Wu T., Wang X., Cheng J., Liang X., Li Y., Chen M., Kong L., Tang M. (2022). Nitrogen-Doped Graphene Quantum Dots Induce Ferroptosis through Disrupting Calcium Homeostasis in Microglia. Part. Fibre Toxicol..

[29] Chen Y., Rivers-Auty J., Cricǎ L.E., Barr K., Rosano V., Arranz A.E., Loret T., Spiller D., Bussy C., Kostarelos K. (2021). Dynamic Interactions and Intracellular Fate of Label-Free, Thin Graphene Oxide Sheets within Mammalian Cells: Role of Lateral Sheet Size. Nanoscale Adv..

[30] Pandit S., Gaska K., Kádár R., Mijakovic I. (2021). Graphene-Based Antimicrobial Biomedical Surfaces. ChemPhysChem.

[31] Linklater D.P., Baulin V.A., Juodkazis S., Ivanova E.P. (2018). Mechano-Bactericidal Mechanism of Graphene Nanomaterials. Interface Focus.

[32] Pierini F., Lanzi M., Nakielski P., Pawłowska S., Zembrzycki K., Kowalewski T.A. (2016). Electrospun Poly(3-Hexylthiophene)/Poly(Ethylene Oxide)/Graphene Oxide Composite Nanofibers: Effects of Graphene Oxide Reduction. Polym. Adv. Technol..

[33] Shin Y.C., Lee J.H., Jin O.S., Kang S.H., Hong S.W., Kim B., Park J.C., Han D.W. (2015). Synergistic Effects of Reduced Graphene Oxide and Hydroxyapatite on Osteogenic Differentiation of MC3T3-E1 Preosteoblasts. Carbon.

[34] Bebber C.M., Müller F., Clemente L.P., Weber J., von Karstedt S. (2020). Ferroptosis in Cancer Cell Biology. Cancers.

[35] Huang G., Chen H., Dong Y., Luo X., Yu H., Moore Z., Bey E.A., Boothman D.A., Gao J. (2013). Superparamagnetic Iron Oxide Nanoparticles: Amplifying Ros Stress to Improve Anticancer Drug Efficacy. Theranostics.

[36] Yu X., Zhang Y.-C., Yang X., Huang Z., Zhang T., Yang L., Meng W., Liu X., Gong P., Forni A. (2022). Bonsai-Inspired AIE Nanohybrid Photosensitizer Based on Vermiculite Nanosheets for Ferroptosis-Assisted Oxygen Self-Sufficient Photodynamic Cancer Therapy. Nano Today.

[37] Pan W.L., Tan Y., Meng W., Huang N.H., Zhao Y.B., Yu Z.Q., Huang Z., Zhang W.H., Sun B., Chen J.X. (2022). Microenvironment-Driven Sequential Ferroptosis, Photodynamic Therapy, and Chemotherapy for Targeted Breast Cancer Therapy by a Cancer-Cell-Membrane-Coated Nanoscale Metal-Organic Framework. Biomaterials.

[38] Li Q., Su R., Bao X., Cao K., Du Y., Wang N., Wang J., Xing F., Yan F., Huang K. (2022). Glycyrrhetinic Acid Nanoparticles Combined with Ferrotherapy for Improved Cancer Immunotherapy. Acta Biomater..

[39] Zheng Y., Li X., Dong C., Ding L., Huang H., Zhang T., Chen Y., Wu R. (2022). Ultrasound-Augmented Nanocatalytic Ferroptosis Reverses Chemotherapeutic Resistance and Induces Synergistic Tumor Nanotherapy. Adv. Funct. Mater..

[40] Pan X., Qi Y., Du Z., He J., Yao S., Lu W., Ding K., Zhou M. (2021). Zinc Oxide Nanosphere for Hydrogen Sulfide Scavenging and Ferroptosis of Colorectal Cancer. J. Nanobiotechnol..

[41] Xu R., Yang J., Qian Y., Deng H., Wang Z., Ma S., Wei Y., Yang N., Shen Q. (2021). Ferroptosis/Pyroptosis Dual-Inductive Combinational Anti-Cancer Therapy Achieved by Transferrin Decorated NanoMOF. Nanoscale Horizons.

[42] Hsieh C.-H., Hsieh H.-C., Shih F.-S., Wang P.-W., Yang L.-X., Shieh D.-B., Wang Y.-C. (2021). An Innovative NRF2 Nano-Modulator Induces Lung Cancer Ferroptosis and Elicits an Immunostimulatory Tumor Microenvironment. Theranostics.

[43] Li Z., Wu X., Wang W., Gai C., Zhang W., Li W., Ding D. (2021). Fe(II) and Tannic Acid-Cloaked MOF as Carrier of Artemisinin for Supply of Ferrous Ions to Enhance Treatment of Triple-Negative Breast Cancer. Nanoscale Res. Lett..

[44] Tang H., Li C., Zhang Y., Zheng H., Cheng Y., Zhu J., Chen X., Zhu Z., Piao J.G., Li F. (2020). Targeted Manganese Doped Silica Nano GSH-Cleaner for Treatment of Liver Cancer by Destroying the Intracellular Redox Homeostasis. Theranostics.

[45] Sang M., Luo R., Bai Y., Dou J., Zhang Z., Liu F., Xu J., Liu W., Feng F. (2019). Mitochondrial Membrane Anchored Photosensitive Nano-Device for Lipid Hydroperoxides Burst and Inducing Ferroptosis to Surmount Therapy-Resistant Cancer. Theranostics.

[46] Rani Aluri E., Gannon E., Singh K., Kolagatla S., Kowiorski K., Shingte S., McKiernan E., Moloney C., McGarry K., Jowett L. (2022). Graphene Oxide Modulates Inter-Particle Interactions in 3D Printable Soft Nanocomposite Hydrogels Restoring Magnetic Hyperthermia Responses. J. Colloid Interface Sci..

[47] Nasiłowska B., Bogdanowicz Z., Hińcza K., Mierczyk Z., Góźdź S., Djas M., Kowiorski K., Bombalska A., Kowalik A. (2020). Graphene Oxide Aerosol Deposition and Its Influence on Cancer Cells. Preliminary Results. Materials.

[48] EP 2778128 B1 20160302—Method of Thermal Reduction of Graphene OXIDE. https://data.epo.org/gpi/EP2778128B1-METHOD-OF-THERMAL-REDUCTION-OF-GRAPHENE-OXIDE.

[49] EP 2653445 B1 20171011—The Method of Graphene Oxide Chemical Reduction. https://data.epo.org/gpi/EP2653445B1-The-method-of-graphene-oxide-chemical-reduction.

[50] Xiaoli F., Qiyue C., Weihong G., Yaqing Z., Chen H., Junrong W., Longquan S. (2020). Toxicology Data of Graphene-Family Nanomaterials: An Update. Arch. Toxicol..

[51] Wang S., Cole I.S., Li Q. (2016). The Toxicity of Graphene Quantum Dots. RSC Adv..

[52] Mittal S., Kumar V., Dhiman N., Chauhan L.K.S., Pasricha R., Pandey A.K. (2016). Physico-Chemical Properties Based Differential Toxicity of Graphene Oxide/Reduced Graphene Oxide in Human Lung Cells Mediated through Oxidative Stress. Sci. Rep..

[53] Nasirzadeh N., Azari M.R., Rasoulzadeh Y., Mohammadian Y. (2019). An Assessment of the Cytotoxic Effects of Graphene Nanoparticles on the Epithelial Cells of the Human Lung. Toxicol. Ind. Health.

[54] Jagiełło J., Chlanda A., Baran M., Gwiazda M., Lipińska L. (2020). Synthesis and Characterization of Graphene Oxide and Reduced Graphene Oxide Composites with Inorganic Nanoparticles for Biomedical Applications. Nanomaterials.

[55] Desante G., Labude N., Rütten S., Römer S., Kaufmann R., Zybała R., Jagiełło J., Lipińska L., Chlanda A., Telle R. (2021). Graphene Oxide Nanofilm to Functionalize Bioinert High Strength Ceramics. Appl. Surf. Sci..

[56] Bullock C.J., Bussy C. (2019). Biocompatibility Considerations in the Design of Graphene Biomedical Materials. Adv. Mater. Interfaces.

[57] Liu S., Zeng T.H., Hofmann M., Burcombe E., Wei J., Jiang R., Kong J., Chen Y. (2011). Antibacterial Activity of Graphite, Graphite Oxide, Graphene Oxide, and Reduced Graphene Oxide: Membrane and Oxidative Stress. ACS Nano.

[58] Jagiełło J., Sekuła-Stryjewska M., Noga S., Adamczyk E., Dźwigońska M., Kurcz M., Kurp K., Winkowska-Struzik M., Karnas E., Boruczkowski D. (2019). Impact of Graphene-Based Surfaces on the Basic Biological Properties of Human Umbilical Cord Mesenchymal Stem Cells: Implications for Ex Vivo Cell Expansion Aimed at Tissue Repair. Int. J. Mol. Sci..

[59] Oberbek P., Bolek T., Chlanda A., Hirano S., Kusnieruk S., Rogowska-Tylman J., Nechyporenko G., Zinchenko V., Swieszkowski W., Puzyn T. (2018). Characterization and Influence of Hydroxyapatite Nanopowders on Living Cells. Beilstein J. Nanotechnol..

[60] Dulinska-Molak I., Chlanda A., Li J., Wang X., Bystrzejewski M., Kawazoe N., Chen G., Swieszkowski W. (2018). The Influence of Carbon-Encapsulated Iron Nanoparticles on Elastic Modulus of Living Human Mesenchymal Stem Cells Examined by Atomic Force Microscopy. Micron.

[61] Mattheolabakis G., Mikelis C.M. (2019). Nanoparticle Delivery and Tumor Vascular Normalization: The Chicken or The Egg?. Front. Oncol..

[62] Zuchowska A., Dabrowski B., Jastrzebska E., Mazurkiewicz-Pawlicka M., Malolepszy A., Stobinski L., Trzaskowski M., Brzozka Z. (2020). Cytotoxic Properties of Graphene Derivatives Depending on Origin and Type of Cell Line. J. Mater. Res..

[63] Fiorillo M., Verre A.F., Iliut M., Peiris-Pagés M., Ozsvari B., Gandara R., Cappello A.R., Sotgia F., Vijayaraghavan A., Lisanti M.P. (2015). Graphene Oxide Selectively Targets Cancer Stem Cells, across Multiple Tumor Types: Implications for Non-Toxic Cancer Treatment, via “Differentiation-Based Nano-Therapy”. Oncotarget.

[64] Perini G., Palmieri V., Ciasca G., De Spirito M., Papi M. (2020). Unravelling the Potential of Graphene Quantum Dots in Biomedicine and Neuroscience. Int. J. Mol. Sci..

[65] Su W.C., Ku B.K., Kulkarni P., Cheng Y.S. (2016). Deposition of Graphene Nanomaterial Aerosols in Human Upper Airways. J. Occup. Environ. Hyg..

[66] Ou L., Song B., Liang H., Liu J., Feng X., Deng B., Sun T., Shao L. (2016). Toxicity of Graphene-Family Nanoparticles: A General Review of the Origins and Mechanisms. Part. Fibre Toxicol..

[67] Mukherjee S.P., Bottini M., Fadeel B. (2017). Graphene and the Immune System: A Romance of Many Dimensions. Front. Immunol..

[68] Jia P.P., Sun T., Junaid M., Xiong Y.H., Wang Y.Q., Liu L., Pu S.Y., Pei D.S. (2019). Chronic Exposure to Graphene Oxide (GO) Induced Inflammation and Differentially Disturbed the Intestinal Microbiota in Zebrafish. Environ. Sci. Nano.

[69] Burnett M., Abuetabh Y., Wronski A., Shen F., Persad S., Leng R., Eisenstat D., Sergi C. (2020). Graphene Oxide Nanoparticles Induce Apoptosis in Wild-Type and CRISPR/Cas9-IGF/IGFBP3 Knocked-out Osteosarcoma Cells. J. Cancer.

[70] Zhang X., Yin J., Peng C., Hu W., Zhu Z., Li W., Fan C., Huang Q. (2011). Distribution and Biocompatibility Studies of Graphene Oxide in Mice after Intravenous Administration. Carbon.

[71] Fadeel B., Bussy C., Merino S., Vázquez E., Flahaut E., Mouchet F., Evariste L., Gauthier L., Koivisto A.J., Vogel U. (2018). Safety Assessment of Graphene-Based Materials: Focus on Human Health and the Environment. ACS Nano.

